# Rapid capture and detection of ostreid herpesvirus-1 from Pacific oyster *Crassostrea gigas* and seawater using magnetic beads

**DOI:** 10.1371/journal.pone.0205207

**Published:** 2018-10-03

**Authors:** Anna Toldrà, Karl B. Andree, Edgar Bertomeu, Ana Roque, Noèlia Carrasco, Ignasi Gairín, M. Dolores Furones, Mònica Campàs

**Affiliations:** IRTA, Ctra., Sant Carles de la Ràpita, Tarragona, Spain; Hellenic Center for Marine Research, GREECE

## Abstract

Ostreid herpesvirus-1 (OsHV-1) has been involved in mass mortality episodes of Pacific oysters *Crassostrea gigas* throughout the world, causing important economic losses to the aquaculture industry. In the present study, magnetic beads (MBs) coated with an anionic polymer were used to capture viable OsHV-1 from two types of naturally infected matrix: oyster homogenate and seawater. Adsorption of the virus on the MBs and characterisation of the MB-virus conjugates was demonstrated by real-time quantitative PCR (qPCR). To study the infective capacity of the captured virus, MB-virus conjugates were injected in the adductor muscle of naïve spat oysters, using oyster homogenate and seawater without MBs as positive controls, and bare MBs and sterile water as negative controls. Mortalities were induced after injection with MB-virus conjugates and in positive controls, whereas no mortalities were recorded in negative controls. Subsequent OsHV-1 DNA and RNA analysis of the oysters by qPCR and reverse transcription qPCR (RT-qPCR), respectively, confirmed that the virus was the responsible for the mortality event and the ability of the MBs to capture viable viral particles. The capture of viable OsHV-1 using MBs is a rapid and easy isolation method and a promising tool, combined with qPCR, to be applied to OsHV-1 detection in aquaculture facilities.

## Introduction

Pacific oyster *Crassostrea gigas* represents the most important species for global aquaculture production of oysters. However, infectious diseases often result in massive losses and their management remains a major concern [1, 2]. During recent years, massive mortality outbreaks of *C*. *gigas* have been reported in many European countries (e.g. France, Spain, Italy and Ireland), New Zealand, Japan, USA and Australia [3–8]. These mortalities mostly take place during the spring/summer period, when the seawater temperature reaches 16°C [9–11], and have been associated to the detection of ostreid herpesvirus type 1 (OsHV-1). OsHV-1 is an enveloped virus of double-stranded DNA with an icosahedral structure and a diameter size of about 120 nm. It belongs to the *Malacoherpesviridae* family, and is the only member of the genus *Ostreavirus* [12].

The economic importance of *C*. *gigas* oyster aquaculture has favoured the development of diagnostic tools for the detection of OsHV-1 including conventional PCR [13, 14], *in situ* hybridization [15] and immunohistochemistry [16]. However, these methods are time consuming, laborious and qualitative, limiting its practical use in disease prevention and control. Recently, more rapid, specific and sensitive methods such as real-time quantitative PCR (qPCR) have been described and applied to OsHV-1 detection [17]. Despite being routinely applied for OsHV-1 quantification, qPCR does not specify whether DNA correspond to viable viruses, which are necessary to cause viral infection [18]. Addressing the infectivity of viruses is essential to understand their persistence in the environment and the estimation of the risk of transmission. Presently, the only way to assess the infectivity of OsHV-1 involves experimental infections in bivalves, further evaluation of oyster mortality and/or DNA and mRNA analysis by qPCR and reverse transcription qPCR (RT-qPCR), respectively. However, infectivity assays are difficult and greatly depend on the assay conditions (e.g. age, physiology and genetics of oysters). The combination of qPCR with capture systems able to separate between viable and non-viable virus particles from the environmental matrix may provide a feasible approach to directly detect viable virus particles, as an alternative to the use of infectivity assays.

On the other hand, obtaining OsHV-1 viral particles to develop and validate diagnostic tools and for other research purposes (e.g. antibody development, functional studies and sequencing) is nowadays of utmost interest. Because no stable bivalve cell lines are available, the virus cannot be cultured and can only be obtained from infected bivalves [19, 20]. A promising approach has been recently described, which uses haemocytes collected from spat oysters to develop *in vitro* models and to study host/virus interactions [21]. Additionally, subsequent virus isolation and purification involves high speed ultracentrifugation, which is time consuming and requires very expensive equipment. Although polyethylene glycol (PEG) precipitation has also been used for this purpose, it may be incompatible with virus detection techniques. Furthermore, both ultracentrifugation and PEG precipitation often decrease the infectivity of the isolated virus particles [22]. As a result, there is a strong interest in developing rapid and efficient isolation/purification methods as well as new diagnostic tools for OsHV-1.

An alternative approach to isolate viruses is the use of magnetic beads (MBs) coated with antibodies or organic chemicals that efficiently bind viral particles. Poly (methyl vinyl ether-maleic anhydrate) [poly (MVE-MA)] and its derivatives are copolymers with molecular and physicochemical characteristics that allow them to be used as bioadhesives [22]. In this sense, MBs coated with anionic poly (MVE-MA) have been used to capture different types of viruses. These include some enveloped viruses such as human immunodefiency virus (HIV) [23], respiratory syncytial virus (RSV) [24] or influenza virus [25], as well as non-enveloped viruses like adenovirus [26]. Although this technology has a broad potential for the isolation of diverse viruses, the binding capacity of the MBs has been reported to vary depending on the virus type, and even fails to effectively bind some virus types [26, 27]. Thus, its universal applicability in all virus types has not been proven. The exact mode of interaction between the virus particle and the anionic MB remains unclear, but it seems that electrostatic, hydrophilic and hydrophobic interactions are involved [24, 26]. Several parameters such as physical characteristics of the virus (isoelectric point, particle size), membrane properties (electric charge, hydrophobicity) and sample characteristics (pH, ionic strength) may influence virus adsorption [24]. The ability of the MBs to capture virus has been demonstrated in only a few matrices, mainly cell culture media and phosphate-buffered saline, and its application in biological samples has not been tested yet [26, 28]. Compared to traditional isolation techniques, the MB-mediated capture method has several advantages: i) it is fully compatible with subsequent conventional detection techniques (e.g. PCR, ELISA), ii) it is rapid, inexpensive and easy to perform, and iii) it is able to preserve the infective capacity of the isolated virus.

**Fig 1:**
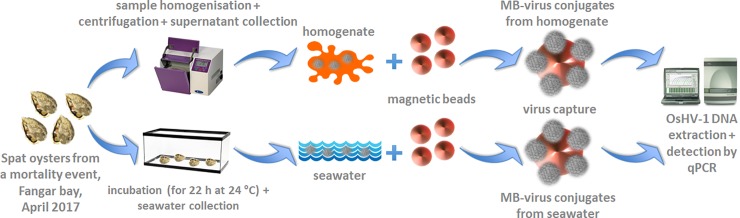
Schematic representation of the protocol for the capture of viable OsHV-1 from the homogenate and the seawater using anionic MBs and subsequent virus detection.

The aim of the present work was to investigate the use of MBs coated with an anionic polymer to capture viable OsHV-1 particles from two types of samples: oyster homogenate and seawater (Fig 1). Adsorption of OsVH-1 on the MBs was assessed by qPCR analysis, and characterisation of the MB-virus conjugates was performed. Afterwards, MB-virus conjugates were injected into naïve spat oysters. The ability of the captured virus to produce an active infection in oysters was evaluated through mortality monitoring as well as DNA and RNA analysis of oysters by qPCR and RT-qPCR, respectively.

## Materials and methods

IRTA is a public research institute owned by the Government of Catalonia and ascribed to the Department of Agriculture. No specific permissions were required for this location/activity. The study did not involve endangered or protected species.

### Reagents

Anionic polymer-coated magnetic beads (viro-adembeads) with a diameter of 300 nm were purchased from Ademtech (Pessac, France). Qiagen DNeasy Blood and Tissue kit, Qiagen RNeasy Mini kit, SYBR Green dye, SuperScript First-Strand cDNA Synthesis kit, RNA*later* and custom primers were supplied by Thermo Fisher Scientific (Madrid, Spain).

### Infected material

*C*. *gigas* spat oysters naturally infected with OsHV-1 were collected from Fangar Bay (NW Mediterranean Sea, N 40˚46’27.62” E 0˚44’12.27”) during a mortality event on April 2017, and OsHV-1 prevalence was evaluated by qPCR following the protocol described in section “OsHV-1 DNA extraction and qPCR analysis, Materials and methods”. These animals were used to prepare two types of samples: (1) Homogenate: 30 oysters (~23 mm in length) were mixed with 20 mL of UV-treated seawater and homogenised using a stomacher for 1 min at maximum speed; after centrifugation (1,000 g; 10 min), the supernatant was collected. (2) Seawater: 30 oysters were placed in a 500-mL UV-treated seawater tank and maintained at 22°C; after 22h, the seawater was collected. Both sets of samples were stored at 4°C until use.

### Capture of OsHV-1 by MBs

Virus capture by MBs was performed following the company’s instructions with some modifications. Briefly, MB-virus conjugates were prepared as follows: (1) 10 μL of MB suspension were transferred to a tube and washed three times with binding buffer (supplied with the MBs); for the washing steps, the tube was placed on the magnetic separation stand and the supernatant was removed; (2) 100 μL of infected material (homogenate or seawater) was added and incubated for 20 min at room temperature with slow tilt-rotation; (3) MB-virus conjugates were washed once with washing buffer (100 mM potassium phosphate, 0.05% v/v Tween-20, pH 7.4) (three times to evaluate the necessity to include several washing steps), resuspended in 100 μL of sterile water and subjected to DNA extraction (and subsequent qPCR analysis) or oyster infection. When other amounts of MB were used, volumes were adjusted proportionally. Characterisation of the conjugates was performed using serial dilutions of the homogenate (pure and 4-fold dilutions) and serial dilutions of MBs (4-fold dilutions; from 160 μL to 0.625 μL) while maintaining a constant amount of MB (10 μL) and using non-diluted homogenate, respectively. Conjugates were prepared and analysed in triplicate.

### Infectivity experiment

Experimental infections were performed using naïve spat *C*. *gigas* oysters (~25 mm in length) from IRTA hatchery, which had been previously found to be negative for the presence of OsHV-1 DNA by qPCR following the protocol described in section “DNA extraction and qPCR analysis, Materials and methods”. The experimental design included 6 treatments: MB-virus conjugates from the homogenate (7.7 x 10^1^ DNA copies/μL), MB-virus conjugates from the seawater (2.9 DNA copies/μL), bare MBs, homogenate (1.1 x 10^4^ DNA copies/μL), seawater (4.2 x 10^2^ DNA copies/μL) and sterile water. Oysters were placed on ice and, once relaxed, 50 μL of the corresponding sample were injected into the adductor muscle of each animal using a syringe. Oysters were then placed in 6 aquaria (26–30 oysters per aquarium/treatment) containing 10 L of UV-treated seawater at 22°C and an independent aeration source. Oysters were fed every 2 days for 11 days with a mix of *Isochrysis sp*., *Tetraselmis sp*. and *Chaetoceros gracilis* and water parameters (pH, salinity, oxygen concentration) were monitored. Mortality was checked daily, dead and moribund oysters being removed from aquaria. At day 11, living oysters were also collected. Oyster tissues (combinations of mantle, gill, digestive gland and adductor muscle) were frozen at -80°C until DNA extraction. Tissues of some moribund and living oysters (3 per treatment with infected material or MB-virus conjugates) were also stored at -80°C with 700 μL of RNA*later* for further RNA extraction.

### OsHV-1 DNA extraction and qPCR analysis

Total DNA was extracted using the Qiagen DNeasy Blood and Tissue kit, according to the manufacturer’s protocols with some adjustments. For the infected material, 100 μL of homogenate or seawater were mixed with 100 μL of lysis buffer (1 M NaCl, 70 mM Tris, 30 mM EDTA, pH 8.6), 20 μL of proteinase K and 200 μL of AL buffer. After 1 h at 56°C, the content was mixed with 200 μL of ethanol and DNA extraction was carried out using spin columns. Elution was performed with 50 μL of AE buffer. The same protocol was used for MB-virus conjugates, the only exception being that MBs were removed after heating using a magnetic separation stand. For oysters, tissue samples were mixed with 180 μL of ATL buffer and 20 μL of proteinase K, and transferred to 2-mL cryotubes containing zirconium glass beads. The mixture was disrupted using a BeadBeater-8 (BioSpec) pulsed for 45 s at full speed and digested overnight at 56°C. Afterwards, 200 μl of buffer AL and 200 μL of ethanol were added, and the contents transferred into the spin columns. Elution was performed with 100 μL of AE buffer. DNA quality and quantity were measured in a NanoDrop 2000 spectrophotometer (Thermo Fisher Scientific). DNA from oysters was diluted to 50 ng/μL. Extracted DNA was stored at -20°C until qPCR analysis.

Detection and quantification of OsHV-1 DNA was carried out by qPCR using the primer pair OsHVDPFor/OsHVDPRev [14]. Reactions were performed using an ABI 7300 thermocycler (Thermo Fisher Scientific) according to the following conditions: 1 cycle of pre-incubation at 95°C for 10 min; 45 cycles of amplification at 95°C for 30 s, 60°C for 1 min s and 72°C for 45 s; and a final step for melting temperature curve analysis at 60°C for 1 min with a gradual increase of temperature (1°C/15 s). Each 20-μL reaction mixture contained 10 μL 2X SYBR Green dye, primers (final concentration 0.5 μM) and 1 μL of extracted DNA. A negative control (without DNA) and a positive control (pure OsHV-1 genomic DNA) were included, and each qPCR reaction was performed in triplicate. Quantification of OsHV-1 DNA copies was carried out using a standard curve based on 10-fold dilutions of OsHV-1 genomic DNA. Results were expressed as total OsHV-1 DNA copies (per 100 μL of homogenate, seawater and MB-virus conjugates, section “Capture of OsHV-1 by MBs, Results and discussion” and section “MB-virus conjugates characterisation, Results and discussion”) or as OsHV-1 DNA copies per ng of total DNA (oyster, section “OsHV-1 DNA quantification in oysters, Results and discussion”).

### OsHV-1 RNA extraction, cDNA synthesis and RT-qPCR analysis

Total RNA extraction from oyster tissue samples was performed with the Qiagen RNeasy Mini kit, using 50 μL of diethylpyrocarbonate (DEPC) treated water for the elution. RNA quality and quantity were determined by spectrophotometry and TAE/formamide agarose electrophoresis. First-stranded cDNA synthesis was carried out using SuperScript First-Strand cDNA Synthesis kit using 2 μg of RNA. To evaluate virus gene expression, primer pairs for 3 OsHV-1 genes (ORF4, ORF16 and ORF42) and one oyster housekeeping gene (Elongation factor 1-alfa, EF1) described by Segarra et al. [29] were used. RT-qPCR reactions were performed in a total volume of 20 μL containing 2 μL of 5-fold diluted cDNA, 10 μL 2X SYBR Green dye and primers (final concentration 0.5 μM). Reactions were performed in triplicate and samples of RNA without reverse transcription were included in order to control absence of genomic DNA contamination. PCR cycling conditions were as follows: 10 min at 95°C, followed by 45 cycles of amplification at 95°C for 15 s, 60°C for 20s. Melting curves were also plotted. Viral gene expression of the 3 viral genes was normalised to EF1 using the formula: Delta Ct = Ct viral ORF–Ct EF1.

### Statistical analysis

Statistical analyses were carried out using SigmaStat software 3.1 (Systat Software Inc., California, USA). The normality of the data was tested using a Shapiro-Wilk test. To evaluate differences between means of viral DNA and RNA amounts quantified in dead/moribund and living oysters, a non-parametrical Mann-Whitney test was conducted. A p-value level of 0.05 was used in all tests to identify differences.

## Results and discussion

### Capture of OsHV-1 by MBs

**Fig 2:**
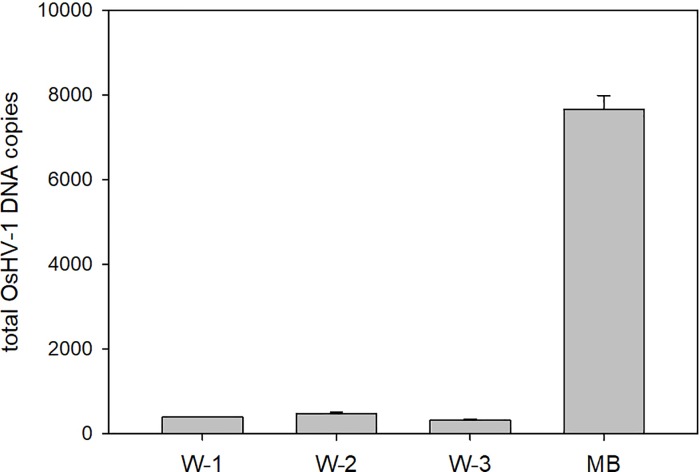
OsHV-1 DNA detected in the supernatant after the first (W-1), second (W-2) and third (W-3) washing steps and in the MB-virus conjugates from homogenate (MB).

Analysis by qPCR of the infected samples revealed the presence of OsHV-1 DNA: 1.1 x 10^6^ ± 3.2 x 10^4^ DNA copies in the homogenate and 4.2 x 10^4^ ± 1.8 x 10^3^ DNA copies in the seawater. To examine the capacity of the MBs to capture the virus from both sample types, the DNA samples extracted from MB-virus conjugates were also analysed by qPCR. OsHV-1 DNA was detected in both conjugates, confirming the ability of the MBs to capture virus from both types of samples: 7.7 x 10^3^ ± 6.3 x 10^2^ DNA copies in the MB-virus conjugates from homogenate and 2.9 x 10^2^ ± 2.5 x 10^1^ DNA copies in the MB-virus conjugates from seawater. It is important to mention that MB-virus conjugates from the homogenate were washed three times and the supernatants from these washing steps were analysed. No differences were obtained between these supernatants, the amount of viral DNA being always below 10% that of the conjugate, hence indicating that the viral DNA measured in the conjugate samples was the result of the MB-virus interaction (Fig 2). In further experiments, only one washing step was included in the protocol. Under the current experimental conditions, capture efficiency from both homogenate and seawater samples was ~1% (percentage calculated using the amount of viral DNA from qPCR data, which only detects viral DNA, and thus may not be reflecting the capacity of the MBs to capture viable virus particles). This low efficiency could be explained by the non-specific adsorption of proteins on the MBs in the case on the homogenate sample, and by the high ionic strength of the seawater, which could screen charge attraction. When compared to other works, high capture efficiencies were achieved using simple matrices (i.e. cell culture media and buffer), whereas the capture of the virus was inhibited when using human blood serum, likely due to the non-specific binding of albumin [26, 28]. In this work, despite the use of complex matrices, OsHV-1 capture was successfully achieved. It should be noted that experimental conditions can be modified (e.g. volume ratios) to improve virus capture efficiency by the beads (see section “MB-virus conjugates characterisation, Results and discussion”).

### MB-virus conjugates characterisation

**Fig 3:**
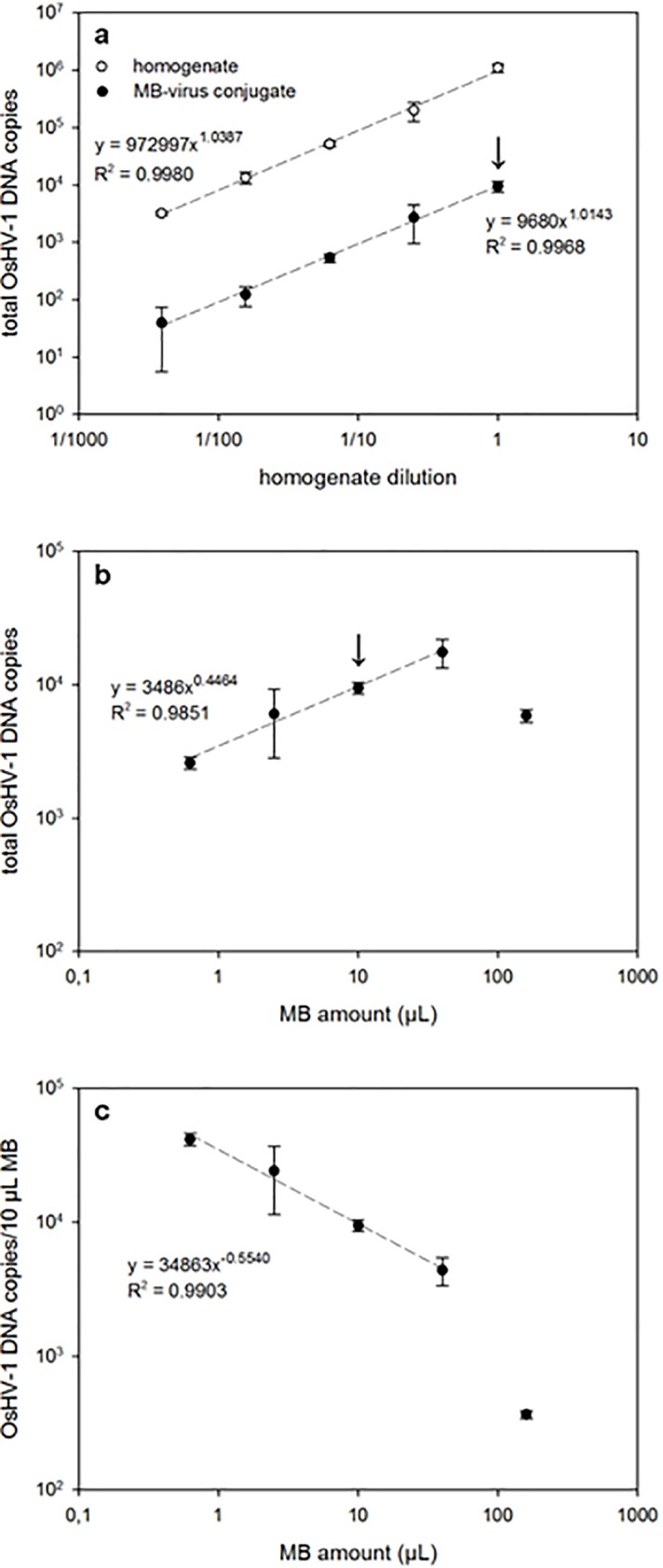
**Calibration curves using 4-fold serial dilutions of homogenate (a) and MBs (b, c) while maintaining MB amount (10 μL) and using non-diluted homogenate, respectively.** Arrows indicate the same MB/homogenate ratio in (a) and (b). Panel (c) is normalised to 10 μL of MBs.

To study the OsHV-1 virus capture by the MBs, two experiments were performed: 1) using serial dilutions of the homogenate and a constant amount of MBs, and 2) using serial dilutions of MBs and non-diluted homogenate. In the first experiment, the amount of OsHV-1 DNA on the MBs decreased with the homogenate dilution (Fig 3A) (no OsHV-1 DNA from MBs was detected when using 1/1,024 homogenate dilution or less). Results show that at each homogenate dilution, there is an equilibrium between the number of virus particles captured by the MBs and the number of free virus particles in solution. Additionally, no saturation of the MBs by virus particles was observed, suggesting that MBs could physically accommodate more virus particles, if present in the homogenate sample. Fig 3B shows the total amount of OsHV-1 DNA on the MBs in the experiment where serial dilutions of MBs were used. Except when 160 μL of MBs were tested (at these conditions steric effects may influence the virus capture efficiency; thus, this point has was not included in the regression), the amount of OsHV-1 DNA on the MBs decreased with the MB dilution. Nevertheless, when the amount of OsHV-1 DNA is normalised to 10 μL of MBs, results indicate that the lower the number of MBs used, the higher the amount of virus particles captured per MB (as before, 160 μL amount was not included in the regression) (Fig 3C). These results clearly indicate that the MB/virus ratio is a key factor in the capture event. Although the highest total amount of viral DNA was recovered using 40 μL of MBs, further experiments were performed with 10 μL of MBs for economic reasons.

### Infectivity experiment

#### Oyster mortality

**Fig 4:**
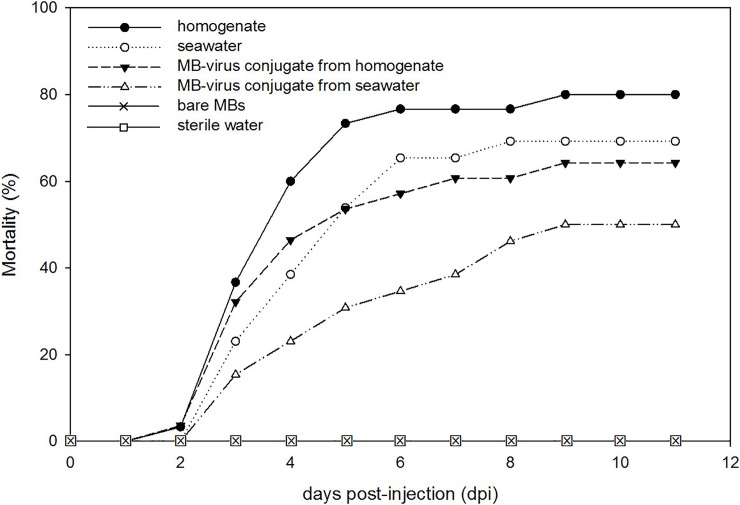
Cumulative mortality of Pacific *Crassostrea gigas* oysters experimentally infected by intramuscular injection with homogenate, seawater, MB-virus conjugates from homogenate and MB-virus conjugates from seawater, bare MBs and sterile water.

No mortality was observed in the aquaria with oysters to which bare MBs or sterile water had been injected in the adductor muscle of naïve spat oysters. The mortality results for the other 4 treatments are shown in Fig 4. Mortality started at the 2^nd^ day post-injection (dpi) for the treatments with homogenate and MB-virus conjugates from homogenate and at the 3^rd^ dpi for the treatments with seawater and MB-virus conjugates from seawater, which may reflect the time needed for viral replication in oyster tissues [30]. At the 9^th^ dpi the cumulative mortality was 80%, 69%, 64% and 50% in the treatments injected with homogenate, seawater, MB-virus conjugates from homogenate and MB-virus conjugates from seawater, respectively, and no more mortalities were recorded afterwards. These results confirm that MBs are able to capture viable OsHV-1 virus particles from both the homogenate and the seawater, and indicate that naïve oysters were successfully infected after injection of MB-virus conjugates. Additionally, a clear dose-response relationship between the number of copies injected (see section “Infectivity experiment, Materials and methods”) and the final cumulative mortality was observed. These mortality rates are in accordance with previous experimental works performed by intramuscular injection using OsHV-1-infected oyster homogenates [20, 31]. The infectivity efficiency was 80% and 72% for the conjugates from homogenate and seawater, respectively, compared to the infected material. Mortalities were detected earlier for the treatment with the MB-virus conjugates from homogenate than for the treatment with seawater (Fig 4). Since lower number of copies were injected with the MB-virus conjugates (see section “Infectivity experiment, Materials and methods”), this earlier mortality may indicate that the presence of MBs could enhance infectivity in oysters. Although the mechanisms by which virus infect cells in the form of anionic poly (MVE-MA) MB-virus conjugates remain unknown, some studies have shown that poly (MVE-MA) derivatives can be used as a vehicle for intracellular transfer, and viruses may be incorporated into cells in the form of conjugates and infect cells [25, 32]. In fact, several phenomena (presence of viable and non-viable virus particles, capture efficiency and/or infectivity potency) may be simultaneously playing a role in this infectivity experiment.

#### OsHV-1 DNA quantification in oysters

**Fig 5:**
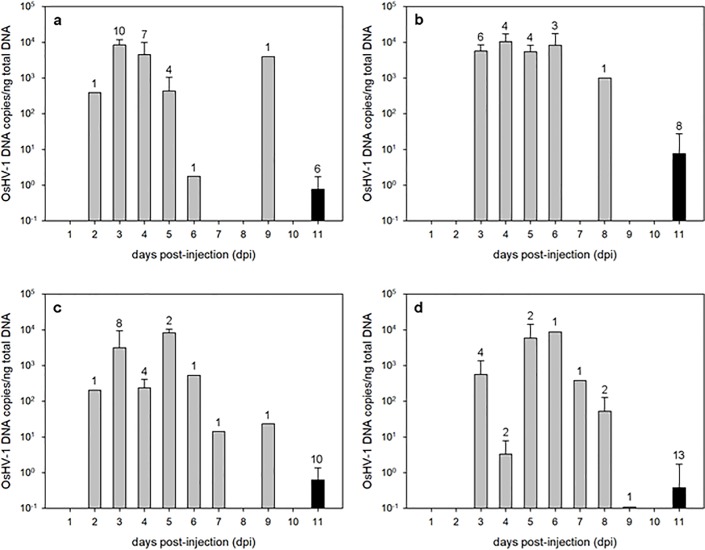
**Viral DNA quantifications by qPCR in dead/moribund (grey bars) and living (black bars) oysters after intramuscular injection of homogenate (a), seawater (b), MB-virus conjugates from homogenate (c) and MB-virus conjugates from seawater (d).** Results (mean ± SD) are expressed as OsHV-1 DNA copies/ng of total DNA. The number above each bar represents the number of oysters analysed.

Analysis of OsHV-1 DNA was performed by qPCR for each individual. OsHV-1 DNA was not detected in oysters to which bare MBs or sterile water had been injected. Viral DNA amounts were above 10^2^ DNA copies/ng total DNA in almost all dead/moribund oysters regardless of the treatment (Fig 5), which suggests that the quantity of viral DNA must reach a threshold in oyster tissue before causing death, as reported in other works [31]. Only a few dead/moribund oysters contained lower viral DNA amounts: 1 oyster injected with homogenate (collected at the 6^th^ pdi), 2 oysters injected with MB-virus conjugates from homogenate (collected at the 7^th^ and 9^th^ pdi) and 4 oysters injected with MB-virus conjugates from seawater (collected at the 4^th^, 8^th^ and 9^th^ pdi). This lower DNA viral content may be explained by DNA degradation processes occurring after death, or individual genetic differences making some individual animals more susceptible [33, 34]. The OsHV-1 DNA amounts found in dead/moribund oysters are in accordance with other studies performed by intramuscular injection [20].

OsHV-1 DNA was also detected in oysters that survived until the end of the experiment. Only 6 oysters injected with MB-virus conjugates from seawater displayed negative results for OsHV-1 DNA detection. For the rest, viral DNA concentrations were below 10^1^ DNA copies/ng total DNA in all treatments, except for 1 oyster injected with seawater (5.7 x 10^1^ DNA copies/ng total DNA). The presence of OsHV-1 DNA has been previously reported in asymptomatic OsHV-1 infected oysters [20, 30, 35]. The presence of living oysters could be attributed to a higher antiviral immune response capacity, which reduces their susceptibility to develop the disease [33–35]. Overall, the amount of viral DNA in dead/moribund oysters was significantly different (p < 0.05) from that found in living oysters in all treatments, which confirms that the oysters were infected by the injection of MB-virus conjugates.

#### OsHV-1 gene expression

**Fig 6:**
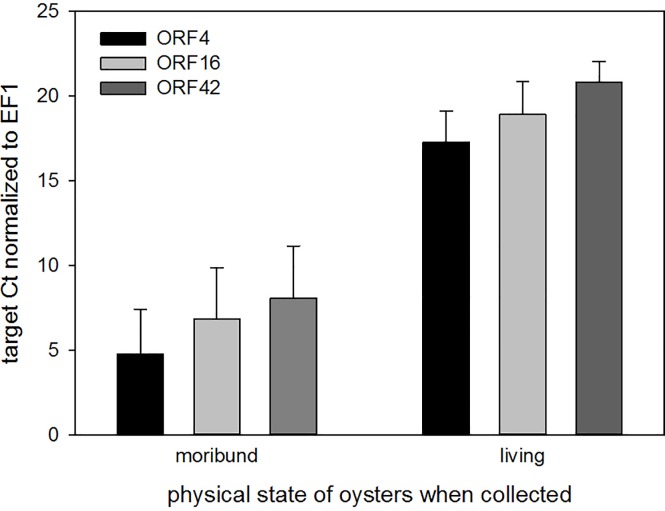
Relative expression of the 3 viral gene transcripts (ORF4, ORAF16, ORF42) in moribund and living oysters. Ct results (mean ± SD) are normalised to EF1. Lower Ct values correspond to earlier detection (higher expression levels) of each transcript. A total of 12 moribund oysters and 12 living oysters were analysed.

The 3 viral ORFs (ORF4, ORF16 and ORF42) selected to study virus gene expression are classified as early genes (expressed before 2h post-injection) and encode proteins with different functions/structures: ORF4 (unknown protein), ORF16 (putative membrane protein) and ORF 42 (putative apoptosis protein). These belongs to different groups/families of genes and were previously studied by Segarra et al. [29]. The oyster host EF1 gene was selected as an endogenous control because it has been frequently used in several marine species [21, 36–38] and it has been reported to be the most stable housekeeping gene in *C*. *gigas* oysters when challenged with OsHV-1 [29]. Moribund animals were collected before mortality occurred to avoid degradation of viral transcripts in dead individuals. Viral RNA expression in living oysters was significantly lower (higher Ct values) (p < 0.05) than in moribund oysters for the 3 viral ORFs (Fig 6). These results indicate an active replication of the virus in moribund oysters and confirm that the mortalities occurring during the time course of the experiment are due to the OsHV-1 infection after injection with MB-virus conjugates.

Summarising, the mortality observed in the oysters to which MB-virus conjugates were injected, together with the high viral DNA loads in dead/moribund oysters and the active viral gene transcription in moribund oysters, clearly demonstrate that OsHV-1 was the causative agent of oyster spat mortality and the ability of the MBs to capture viable viral particles.

## Conclusions

This study reports the use of anionic polymer-coated MBs to capture viable OsHV-1 from both oyster homogenate and seawater samples for the first time. The absorption of the virus on MBs in conjugates from homogenate and seawater was confirmed by qPCR. Furthermore, the viability of the isolated virus particles was demonstrated through experimental infections of naïve oysters and subsequent mortality monitoring as well as DNA and RNA analysis. Further research is needed to elucidate the role of the MBs in the oyster infection, which would be of great interest for pathogenicity studies.

The isolation of viable OsHV-1 is especially important because no stable bivalve cell lines are available to culture the virus and it can only be obtained from naturally infected material. In this sense, another merit of this study is that MBs can be used successfully for virus collection from samples of a complex nature, such as an oyster tissue homogenate, a semi-viscous matrix with a high protein content. Conventional virus isolation techniques are not straightforward, while the method presented herein is simple and provides minimal sample preparation to obtain useful material. Additionally, the isolation of viruses from seawater is key to understanding mechanisms of horizontal transmission of OsHV-1 (direct host-host transmission, or indirect by way of some other vector).

The use of anionic polymer-coated MBs is a rapid, easy and cost-effective strategy to isolate viable OsHV-1 particles from complex matrices, and could be of great utility in research activities such as studies on genetics, physiology, immunology and pathology. Additionally, it could provide a valuable tool when integrated with qPCR or other detection techniques, either to enable viable virus detection (provided that only viable particles are captured by the MBs), to enhance sensitivity by previously concentrating virus particles or to reduce PCR-inhibitory effects by previously purifying the virus. The assay may contribute to increase the knowledge of the OsHV-1 and limit the impact of OsHV-1 infection in the oyster industry.
